# Design optimization of high tibial osteotomy plates using finite element analysis for improved biomechanical effect

**DOI:** 10.1186/s13018-019-1269-8

**Published:** 2019-07-16

**Authors:** Yong-Gon Koh, Jin-Ah Lee, Hwa-Yong Lee, Heoung-Jae Chun, Hyo-Jeong Kim, Kyoung-Tak Kang

**Affiliations:** 1 Department of Orthopaedic Surgery, Joint Reconstruction Center, Yonsei Sarang Hospital, 10 Hyoryeong-ro, Seocho-gu, Seoul, 06698 Republic of Korea; 2 Department of Mechanical Engineering, Yonsei University, 50 Yonsei-ro, Seodaemun-gu, Seoul, 03722 Republic of Korea; 3 Department of Sport and Healthy Aging, Korea National Sport University, 1239 Yangjae-dearo, Songpa-gu, Seoul, 05541 Republic of Korea

**Keywords:** High tibial osteotomy, Design optimization, Finite element analysis

## Abstract

**Background:**

High tibial osteotomy (HTO) is a common treatment for moderate osteoarthritis of the medial compartment in the knee joint by the translation of the force center toward the lateral compartment. However, the stability of a short plate such as Puddu used in this procedure was not as effective as other long plates such as Tomofix. No previous studies have used a rigorous and systematic design optimization method to determine the optimal shape of short HTO plate. Therefore, the purpose of this study is to evaluate the improved biomechanical stability of a short HTO plate by using design optimization and finite element (FE) analysis.

**Methods:**

A FE model of HTO was subjected to physiological and surgical loads in the tibia. Taguchi-style L27 orthogonal arrays were used to identify the most significant factors for optimizing the design parameters. The optimal design variables were calculated using the nondominated sorting genetic algorithm II. Plate and bone stresses and wedge micromotions in the initial and optimized designs were chosen as the comparison indices.

**Results:**

Optimal designed HTO plate showed the decreased micromotions over the initial HTO plate with enhanced plate stability. In addition, increased bone stress and decreased plate stress supported the positive effect on stress shielding compared to initial HTO plate design. The results yielded a new short HTO design while demonstrating the feasibility of design optimization and potential improvements to biomechanical stability in HTO design. The newly developed short HTO plate throughout the optimization and computational simulation showed the improved biomechanical effect as good as the golden standard, TomoFix, does.

**Conclusions:**

This study showed that plate design has a strong influence on the stability after HTO. This study demonstrated that the optimized short plates had low stress shielding effect and less micromotion because of its improvement in biomechanical performances. Our result showed that design optimization is an effective tool for HTO plate design. This information can aid future developments in HTO plate design and can be expanded to other implant designs.

## Introduction

High tibial osteotomy (HTO) is a surgical treatment that realigns the weight-bearing axis to eliminate the effects of unilateral osteoarthritis (OA) in knee joints [1]. Fast and successful treatment to restore normal daily activities in a short period and realignment in the knee joint are primary concerns of HTO surgery. While healing is a complex procedure, post-operative stability with plates and screws for an adequate environment to provide bone healing is also important [2, 3].

It has been well known that there are two types of osteotomies in an HTO system: laterally close and medially open [4, 5]. Medial open osteotomy is more preferable than lateral closing wedge osteotomy because of fewer potential complications such as compartment syndrome, neurological complications, lateral muscle detachment, proximal fibula osteotomy, and limb shortening [6, 7]. The strength of the initial fixation in open wedge osteotomy, and the maintenance of its stability during the healing period, is very important for successful post-operative outcomes and the healing of the osteotomy itself.

There are many variations in fixation systems such as combinations of short or long, locked or unlocked, and with or without a metal block (rectangular or tapered) developed for medial open wedge HTO [8]. Biomechanical and clinical comparative studies are often conducted for surgeons to determine the most appropriate decision from variables in fixation device systems for osteotomy [9–13]. These studies showed that longer plates with locking screws provide better stability in both compression and torsion compared to shorter plates [14, 15].

The TomoFix plate (TomoFix Osteotomy System, DePuy Synthesis, West Chester, PA, USA), which is a long and rigid T-shaped titanium internal with a uniaxial locking fixation system, has been recognized as the gold standard [16]. Despite the advantage of biomechanical stability in the TomoFix plate, it is too large or too long for Asian patients and leads to local irritation [8]. In particular, local irritation was reported as a major complaint after HTO using the plate [17, 18]. Short spacer plates such as the Puddu plate (Arthrex Inc., Naples, FL, USA) result in a low incidence of local irritation, but it has been reported in some studies that it may lead to graft nonunion and implant failure [19, 20]. To overcome these limitations, a low-profile and anatomical short plate needs to be developed for medial open wedge HTO. Newly developed short HTO plate supplements Puddu’s disadvantage in low implant stability and Tomofix’s disadvantage in large size for Asian patients. Finite element (FE) analysis is a computational simulation that evaluates local stresses and strains. Recent studies introduced the potential of FE analysis application in the analysis of HTO [21, 22]. In this technique, three-dimensional (3D) models of bones and implants are transformed into FE models under physiological loads applied for the analysis and prediction of surgical outcomes [21]. Golovakha et al. compared the fixation of four different types with stability profiles for various wedge sizes using FE analysis [23]. Luo et al. compared eight variations in T- and T+I-shaped plate designs using FE analysis [24]. However, to our knowledge, there has been no study to evaluate the optimization of HTO design.

Design optimization was performed using numerical algorithms to determine the most appropriate design. The optimum design provides the best performance with respect to a given performance condition and simultaneously satisfies any design space limitations [25]. Recently, Koh et al. evaluated the biomechanical effect of the TomoFix plate system with respect to different plate designs using a computational simulation [26].

Their results showed that an optimal design demonstrated the feasibility of design optimization and improvements in biomechanical stability for long plate [26]. However, there has been no study for short plate to overcome its disadvantage by design optimization.

Therefore, the purpose of this study was to evaluate the optimum geometry of the HTO with respect to fixation stress and stability by using an FE simulation and optimization analysis. The most significant factors of the stability of a fixation system were evaluated using the design of experiments (DOE) approach in the initial model. Based on these significant factor parameters, a design optimization was performed with these objective functions using the nondominated sorting genetic algorithm II (NSGA-II). The model was focused on the micromotion in wedge and stress on tibia and plate in comparison between initial and optimum short plate design models. In addition, the results from a short HTO plate design and long HTO plate design using TomoFix were compared. We hypothesized that the optimal short plate design plate showed improved stress shielding and stability compared to the initial design.

## Materials and methods

### Development of the three-dimensional model

The right leg of a 36-year-old male was used to represent a geometric tibia model. The medical records for the subject showed neutral lower limb alignment without any anatomical abnormality, previous operation, or arthritis.

3D anatomic structures were reconstructed from the subject’s computed tomography (CT). The medical imaging was performed by using a 64-channel CT scanner (Somatom Sensation 64, Siemens Healthcare, Erlangen, Germany). CT scanning was performed with 0.1-mm slices. Digital CT data was imported into the 3D model reconstruction software Mimics (version 14.0; Materialise, Belgium), which was used to generate a 3D geometrical surface of the tibia at full extension. A 3D knee joint model was established and validated in previous studies [27, 28].

**Fig. 1:**
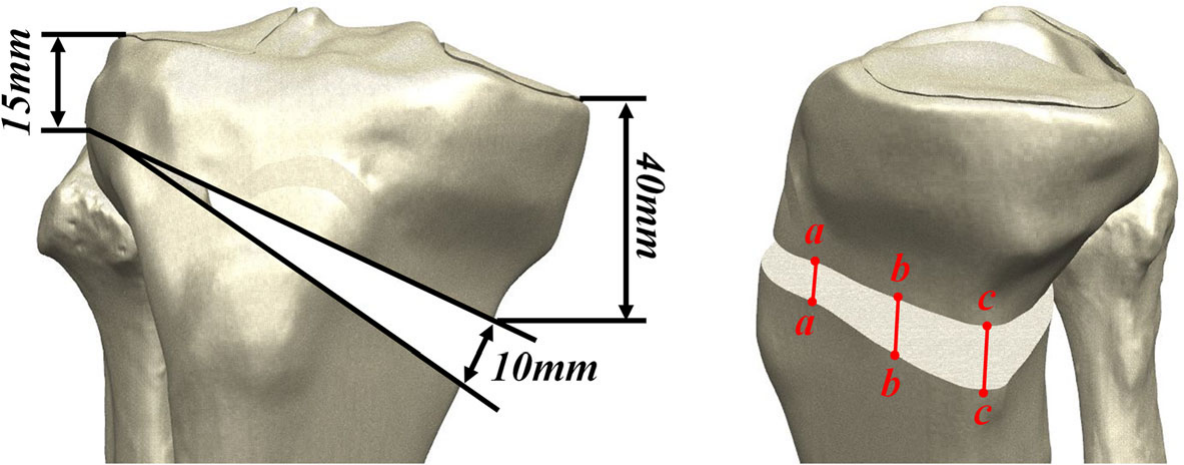
Specifications of the opening wedge HTO used in this study. Three edges *aa*, *bb*, and *cc* along the medial opening were defined to evaluate the height changes in weight-bearing condition

**Fig. 2:**
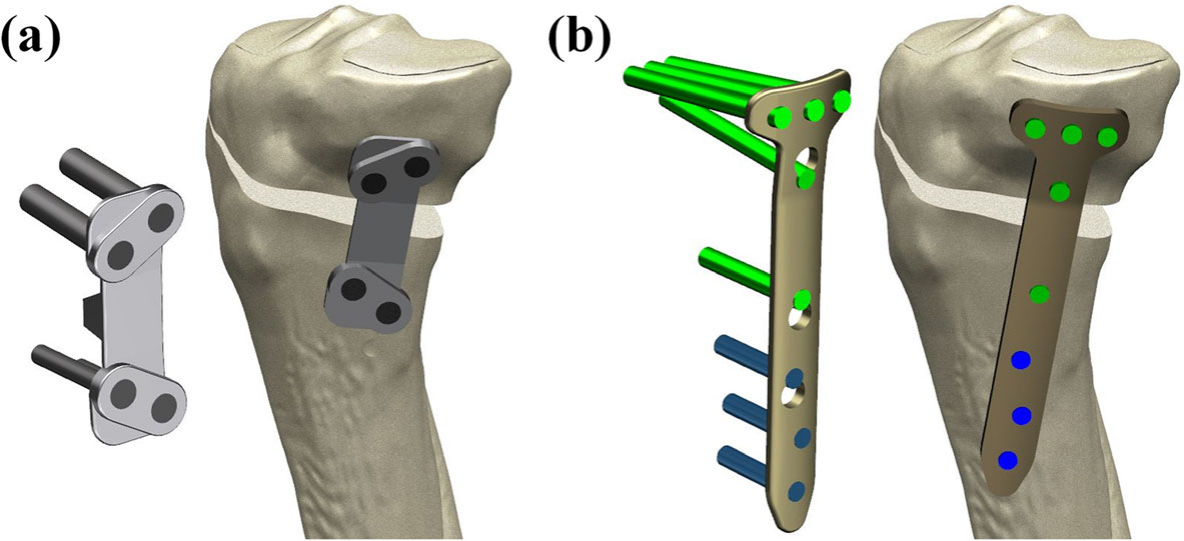
Finite element models for **a** the Puddu plate and **b** the TomoFix plate

The creation of an opening wedge on the medial side was guided by a clinician to simulate an HTO using Unigraphics NX. The knee model was subsequently used to simulate the medial opening wedge HTO with the distal region of the tibia rotated, and opening wedge was simulated in the frontal plane to represent valgus correction angles [29]. The medial opening wedge HTO was simulated so that the loading axis of mechanical axis became lateral 62.5% as Fujisawa et al. suggested [29]. A gap in the opening wedge of 10 mm was simulated by removing a wedge-shaped bone from the proximal tibia (Fig. 1) [21, 22, 24, 30, 31]. There were two types of HTO plate systems used in this study: TomoFix and Puddu, which included the plate and screws. The TomoFix and Puddu plates have a total of eight and four screw holes, respectively, and there were locking head screws between the screw and plate in both plates (Fig. 2) [23].

### Development of the finite element model

The solid model was then imported into Hypermesh (version 10.0; Altair Engineering, Troy, MI, USA) to generate an FE mesh. The bone (cortical and cancellous), plate, and screw were assumed to be linearly elastic, homogeneous, and isotropic. Young’s modulus for the tibia was 17 GPa and 5 GPa for the cortical and cancellous bones, respectively, and Poisson’s ratio was 0.33 [22, 24, 32]. The material properties of the titanium alloy used in the HTO plate were 110 GPa for Young’s modulus and 0.3 for Poisson’s ratio [21]. The geometry was simplified by using thickness constants of 1 mm for the cortical bone of the tibia [33]. A bone graft was removed in the FE analysis to simulate the worst-case scenario for implant loading. The TomoFix and Puddu plates had a length of 112 mm and 17.5 mm, respectively, with the 5 mm and 6 mm of screw diameters. The interfaces between the tibia-plate and the tibia-screw were modeled by using surface-surface contact elements, which allow for separation and slippage [22, 24]. Friction and hard-contact interfaces were modeled, and the friction coefficient was assumed to be 0.3 for tibia-plate and 0.2 for tibia-screw interfaces [21–24, 34]. The locking screws of the HTO plate were simulated to rigidly bond with the plate holes [21–24]. The distal end of the tibia was fixed in all degrees of freedom. The locking screw threads are disregarded for numerical simplification.

**Fig. 3:**
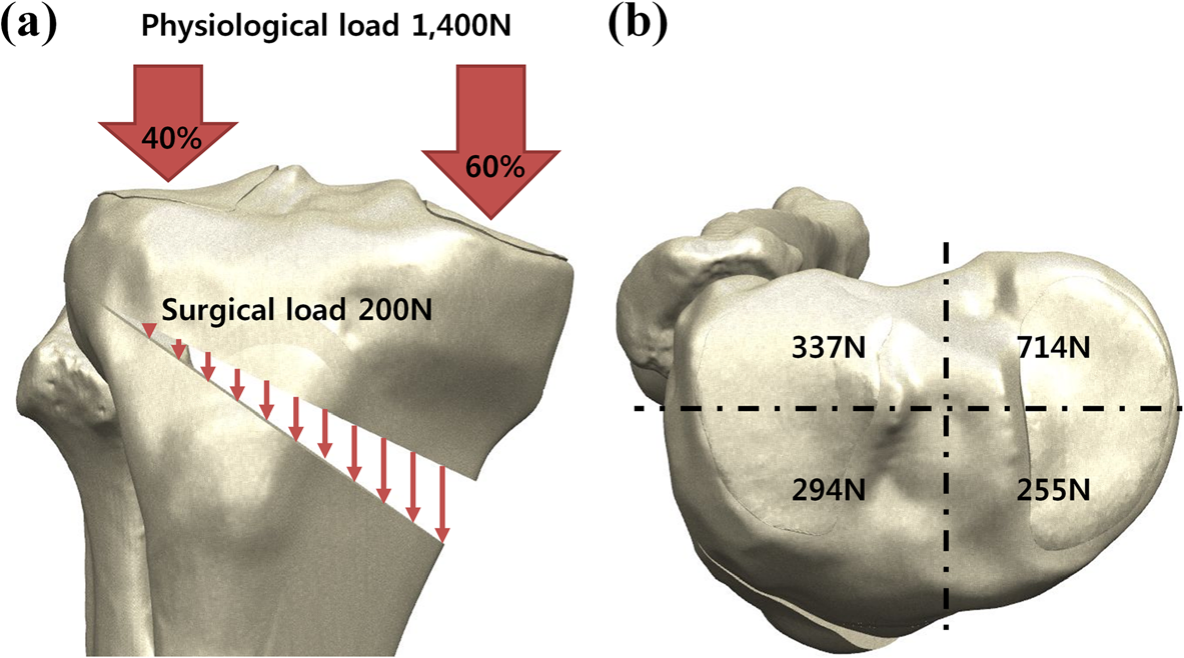
Loading boundary conditions used in this study. **a** Physiological and surgical load. **b** Loads on the four regions of the tibial plateau

Intervention-induced compression during surgery and physiological load surgery was applied to HTO models [22, 24, 35]. The 200-N intervention induced a compressive load that was uniformly exerted onto the tibial opening in a distracted cortex. According to the results from a previous study, restoration of the physiological transfer of the knee load was assumed in this study, and it leads to load repartitions of 40% and 60% on the lateral and medial plateaus, respectively (Fig. 3) [36].

An analysis was conducted using the commercial FE software ABAQUS (version 6.11; Dassault Systèmes, France).

### Parametric and sensitivity analysis using design of experiments

**Fig. 4:**
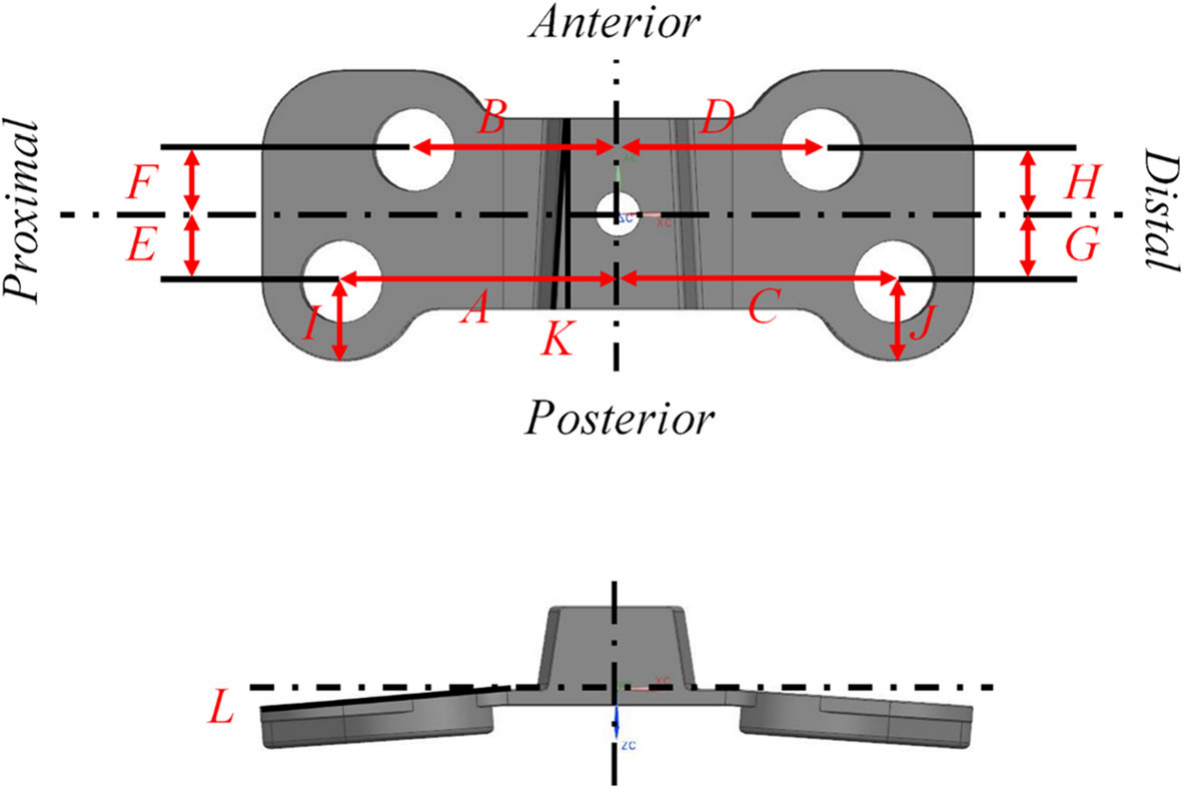
Parameters in geometry of the initial HTO plate

The experimental design was constrained by the relatively large number of factors to be examined, the potential variations of effects, and significant interactions among the factors. In order to analyze the sensitivity of the Puddu plate, a parametric model was developed by setting out the design variables. Based on the model, 12 geometric design variables for the Puddu plate were determined and are depicted in Fig. 4. The thickness of the plate was excluded from the design variables. To assess the results obtained in the parametric study, it is necessary to define a parameter that characterizes the performance of the system for each treatment condition [37].

In the current study, results were analyzed for micromotion in wedge. In DOE, the important factors are determined from all parameters by calculating the effect of the variables on the results. To perform a sensitivity analysis, the L27 orthogonal array was defined. Its details are listed in Table 1. This orthogonal array indicated the range of design according to parameter levels (variation of ± 10% from the initial value) (Table 2).

**Table 1:** Orthogonal array of the HTO used in this study

	*A*	*B*	*C*	*D*	*E*	*F*	*G*	*H*	*I*	*J*	*K*	*L*
#01	1	1	1	1	1	1	1	1	1	1	1	1
#02	1	1	1	1	2	2	2	2	2	2	2	2
#03	1	1	1	1	3	3	3	3	3	3	3	3
#04	1	2	2	2	1	1	1	2	2	2	3	3
#05	1	2	2	2	2	2	2	3	3	3	1	1
#06	1	2	2	2	3	3	3	1	1	1	2	2
#07	1	3	3	3	1	1	1	3	3	3	2	2
#08	1	3	3	3	2	2	2	1	1	1	3	3
#09	1	3	3	3	3	3	3	2	2	2	1	1
#10	2	1	2	3	1	2	3	1	2	3	1	2
#11	2	1	2	3	2	3	1	2	3	1	2	3
#12	2	1	2	3	3	1	2	3	1	2	3	1
#13	2	2	3	1	1	2	3	2	3	1	3	1
#14	2	2	3	1	2	3	1	3	1	2	1	2
#15	2	2	3	1	3	1	2	1	2	3	2	3
#16	2	3	1	2	1	2	3	3	1	2	2	3
#17	2	3	1	2	2	3	1	1	2	3	3	1
#18	2	3	1	2	3	1	2	2	3	1	1	2
#19	3	1	3	2	1	3	2	1	3	2	1	3
#20	3	1	3	2	2	1	3	2	1	3	2	1
#21	3	1	3	2	3	2	1	3	2	1	3	2
#22	3	2	1	3	1	3	2	2	1	3	3	2
#23	3	2	1	3	2	1	3	3	2	1	1	3
#24	3	2	1	3	3	2	1	1	3	2	2	1
#25	3	3	2	1	1	3	2	3	2	1	2	1
#26	3	3	2	1	2	1	3	1	3	2	3	2
#27	3	3	2	1	3	2	1	2	1	3	1	3

**Table 2:** Three levels of parameters with ± 10% from the initial value

	*A*	*B*	*C*	*D*	*E*	*F*	*G*	*H*	*I*	*J*	*K*	*L*
Level 1	19.8	14.4	19.8	14.4	4.5	4.5	4.5	4.5	5.625	5.625	2.7	3.6
Level 2	22.0	16.0	22.0	16.0	5.0	5.0	5.0	5.0	6.250	6.250	3.0	4.0
Level 3	24.2	17.6	24.2	17.6	5.5	5.5	5.5	5.5	6.875	6.875	3.3	4.4

For DOE-based sensitivity analysis, different parameter levels were applied to the results for FE analysis, and they were evaluated using statistical analysis methods such as the analysis of means (ANOM), followed by determination of the important design factors from this analysis. The average micromotion was evaluated using the micromotion at each level. This was important because of the consideration for the sum of squares of deviations (SSD), which was calculated from the difference between the average micromotion and those of each level. A design variable with a high SSD value was considered to be a more sensitive design variable compared with the others.

### Design optimization of short plate

The less important factors were removed through a sensitivity analysis based on DOE, and a design optimization was performed using Isight (version 5.9; Dassault Systèmes). Optimization was conducted by using the nondominated sorting genetic algorithm (NSGA-II). It was proposed in the previous study as a suitable method for solving multi-objective problems [38].

In order to obtain the 100 μm and 150 μm of wedge micromotions at *bb* and *cc*, respectively, and minimal von Mises stress on the plate, the multi-objective function was calculated for the optimal design parameters using NSGA-II. Based on the initial design, a plausible range was determined with respect to position changes of the plate and screw, and the simulations were performed 1000 times with variations at a maximum of ± 50%.

The von Mises stresses for the initial Puddu plate, optimum short plate, and TomoFix plate’s bone, screw, and plate were investigated. In addition, the construction stability for measuring changes in height at edges *aa*, *bb*, and *cc* of the opening was evaluated (Fig. 1).

## Results

### Sensitivity and optimization analysis of design variables for HTO plate

**Fig. 5:**
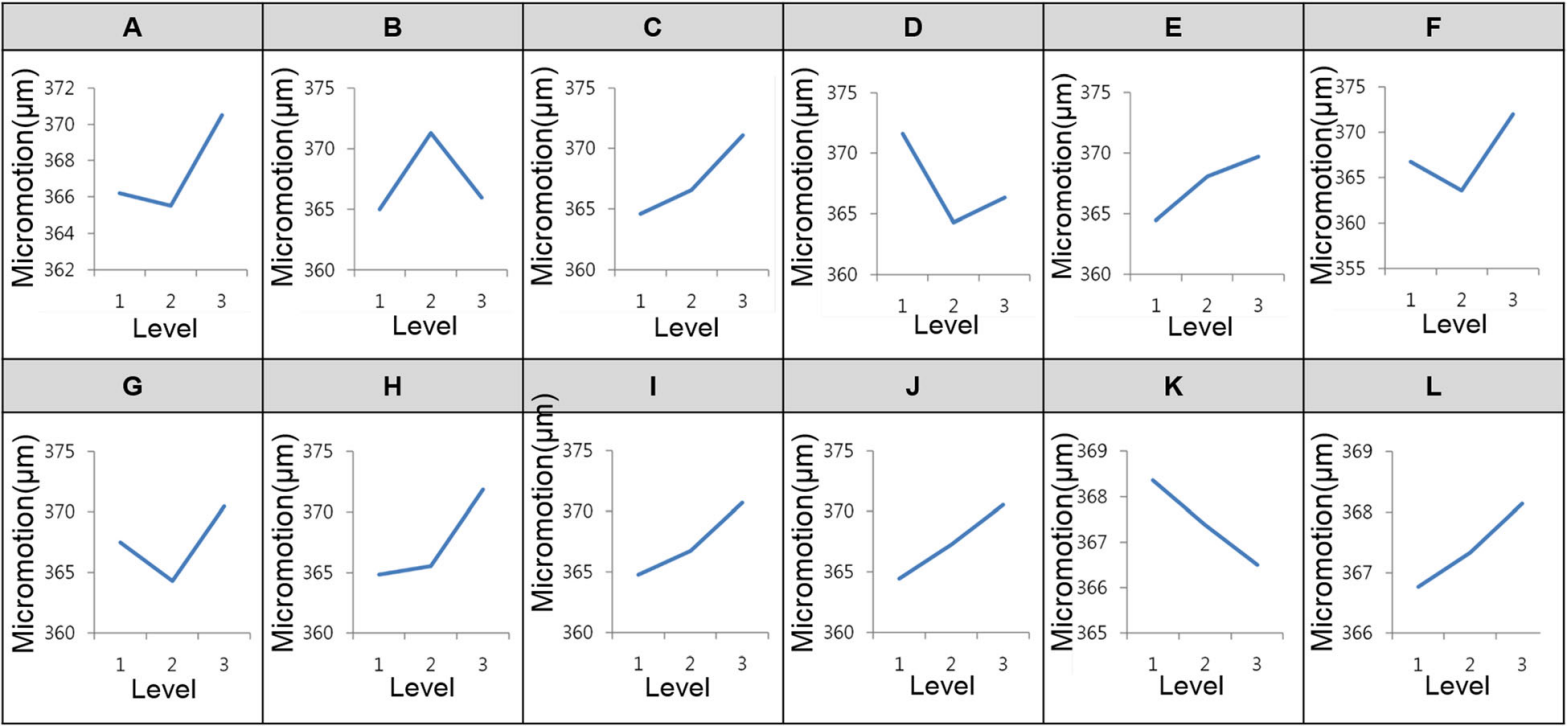
Results of sensitivity analysis of the 12 geometric design variables for the HTO plate

Figure 5 shows the results of a sensitivity analysis of the Puddu plate. These wedge micromotions were evaluated with a large variation from the average value and a large difference based on levels 1, 2, and 3. The high SSD value was calculated, and it was considered an important design variable in the HTO design except for variables *K* and *L*.

*K* is the wedge angle, and *L* is the bending angle along with length direction. Based on this process, the contribution ratio was calculated as a percentage of the total SSD. The sensitivity analysis results are summarized in Table 3. Based on the sensitivity analysis, parameters *K* and *L* were found to have lower contribution ratios compared with the other variables. Therefore, these variables were not considered in the optimization process. Based on the sensitivity results, the optimization process was performed using NSGA-II.

**Table 3:** Results of the sensitivity analysis

	*A*	*B*	*C*	*D*	*E*	*F*
Error sums of squares	14.64171	23.19086	22.08115	28.23486	14.37088	36.0166
Total sum of SS	226.903	226.903	226.903	226.903	226.903	226.903
Contribution ratio (%)	6.45	10.22	9.73	12.44	6.33	15.87
Significant factor	9	4	5	3	10	1
	*G*	*H*	*I*	*J*	*K*	*L*
Error sums of squares	18.89502	29.97681	18.3203	18.48094	1.739026	0.954858
Total sum of SS	226.903	226.903	226.903	226.903	226.903	226.903
Contribution ratio (%)	8.33	13.21	8.07	8.14	0.77	0.42
Significant factor	6	2	8	7	11	12

### Comparison of the stresses on the bone, plate, and screw of the Puddu plate, optimal design short plate, and TomoFix plate

**Fig. 6:**
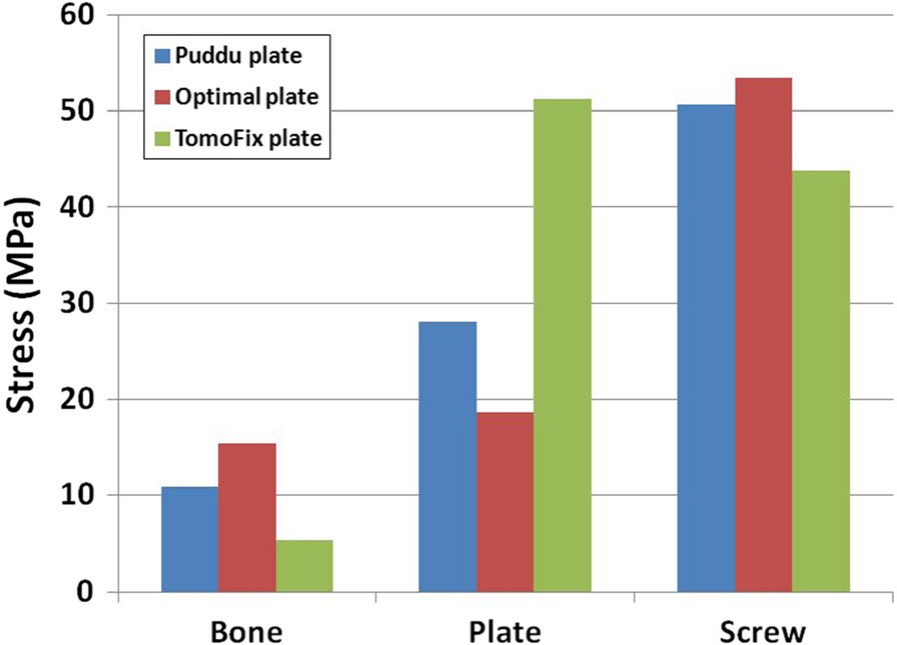
Comparison of the average stresses on the bone, plate, and screw with respect to three difference HTO plates

The stress on the bone was greatest in the optimal design plate, followed by the Puddu plate and the TomoFix plate (Fig. 6). In contrast to the stress on the bone, the stresses on the plate were greatest in the TomoFix plate, followed by the Puddu plate and the optimal design plate (Fig. 6). In addition, the stress on the screw was greatest in the optimal design plate, followed by the Puddu plate and TomoFix plate (Fig. 6). In terms of stresses on the bone and plate, the optimal design plate showed improvement in efficiency with enhanced stress shielding effect compared to the TomoFix plate and Puddu plate. Optimal design stress on the bone and plate increased and decreased, respectively, by 41% and 33% compared with the initial design plate. Increased bone stress and decreased plate stress showed the improvement in stress shielding effect.

**Fig. 7:**
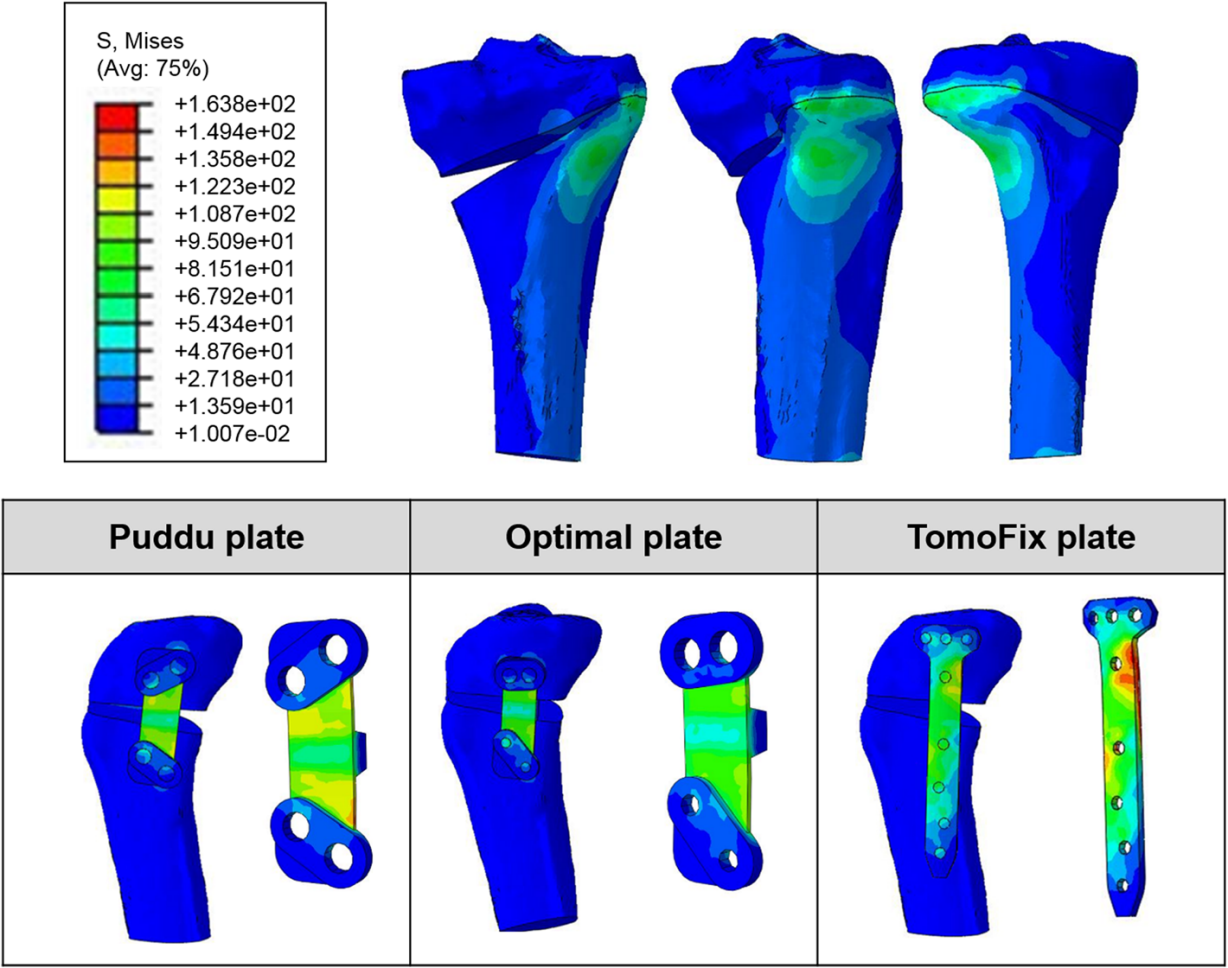
Stress distribution of the bone in the Puddu plate and comparison of the stress distribution on the plate and bone with respect to three difference HTO plates

The stress distribution pattern was similar in all the three plates (Fig. 7). In the model for TomoFix plate, the stress was concentrated in the posterior region of plate and overall region in which opening wedge was supported by plate. Stress concentration was also found in the posterior region for both Puddu plate and optimal design plate, but not in the region of opening wedge because it was supported by a metal block. However, the stress was less concentrated with wider stress distribution in optimal design plate compared to the Puddu plate. For the bone, it was concentrated in the lateral hinge region (Fig. 7).

### Comparison of the micromotion for the Puddu plate, optimal design short plate, and TomoFix plate

**Fig. 8:**
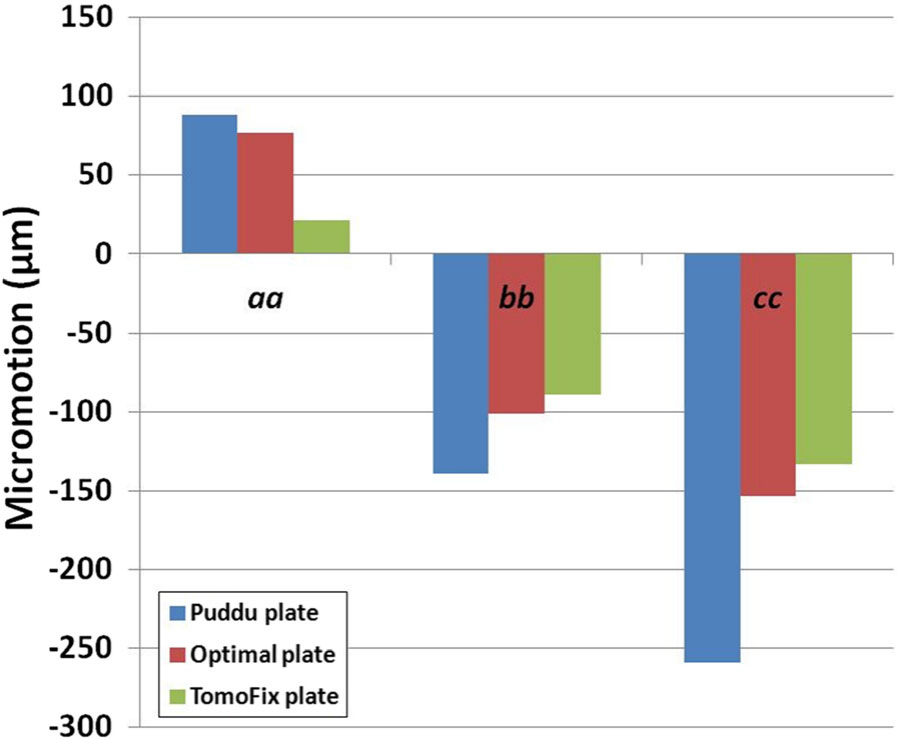
Comparison of the micromotion at edges *aa*, *bb*, and *cc* with respect to different design

The greatest micromotions found in all three different plates were located at edge *cc* (Fig. 8). In addition, tension was exerted at edge *aa*, and compression was exerted at edges *bb* and *cc*. The lowest and greatest micromotions were found in the TomoFix and Puddu plates, respectively.

## Discussion

The most important finding of the current study was that the design of an HTO plate through optimization can lead to biomechanical advantage compared with the initial design plate. The results of the current study were consistent with previous research showing that the TomoFix plate was more rigid and provided better biomechanical effects compared with the Puddu plate [21, 23]. More importantly, this was the primary study proving that a short plate such as a Puddu plate could show functional improvements similar to that of the TomoFix plate throughout the optimization design process. The optimal design plate improved its plate stability because it reduced micromotions compared to the initial design plate. Moreover, load sharing was found as bone stress increased and plate stress decreased.

In several biomechanical comparative studies, the initial stability and fixation strength of specially designed HTO plates were tested [8, 9, 15, 16, 39, 40]. These studies suggested that a rigid long plate provides superior stability during both compression and torsion compared with a short spacer plate. Agneskirchner et al.’s study evaluated an axial load and displacement and reported that less displacement was found when using rigid long medial tibial plate fixators with locking bolts compared with short spacer plates [15].

For long plates, the thickness and material rigidity are the most important factors because a thin and more flexible plate provides less stability compared with a thick and rigid plate [15]. The TomoFix plate provides an internal fixation with locking screw technology. The plate strength in combination with the angular and axial stability of locking screws supports the maximum stability of the osteotomy maintaining the correction [9, 19]. Furthermore, the TomoFix plate is longer and thicker than the Puddu plate. In the second generation, the Puddu plate is 0.5 mm thicker and had locking head screws with cylindrical inserts and a hard screw plate connection compared with the first-generation Puddu plate [23]. In a recent study, the second-generation Puddu plate with a locking screw and thicker plate showed higher stability compared with the first-generation Puddu plate [23].

The purpose of this study was to compare a Puddu plate and TomoFix plate having locking screws, as well as a short optimal design plate developed from the Puddu plate base, by using computational simulations. The wedge sizes were fixed to be 10 mm in three different plate models because a wedge size greater than 10 mm showed a significantly higher overall complication rate based on the failure analysis of a consecutive case series in a previous study [41]. Our results showed that the stress on the bone in the TomoFix plate was lower than that in the Puddu plate. This observation was also reported in an FE analysis in a previous study [21]. That study claimed that a TomoFix that utilizes a locking screw provided a better anchorage than the Puddu plate, thereby displacing the stresses transmitted by body weight [21]. In addition, the angular stability of the locking screw was more rigid than that of the conventional screws used in Puddu plates [21]. The reason was that the TomoFix plate was longer than the Puddu plate, and the thickness and rigidity of the material play an important role. The previous study also supported the fact that a short plate using locking screws did not show as much improvement in stability as a long plate compared with the initial design plate [15]. Moreover, the stress on the plate in the TomoFix plate was 163 MPa, which showed good agreement with the 171 MPa from another study that used FE analysis [22]. Luo et al. reported that the head of the nonlocking screw can be rotated within the plate hole, but the threads of both the screw head and plate hole were tightly locked [22]. The stresses on the screw and plate could be more uniformly distributed and reduced because of contact [21]. Our study showed good agreement with the stresses on the plate and screw in the TomoFix plate given Luo et al.’s results [22].

In any three plates, the stress did not reach the yield strength. In terms of average stress, there was greater stress in the TomoFix plate than in the Puddu plate, but the average stress on the bone was greater in the Puddu plate than in the TomoFix plate. This could be caused by the high rigidity of the TomoFix plate. In other words, the TomoFix plate may cause delayed bone union due to the stress shielding [42].

An interesting finding was that the optimal design short plate showed improvement of biomechanical effect from the current Puddu plate. The stress on the bone and plate increased and decreased by 41% and 33%, respectively, in the optimally designed short plate compared with the Puddu plate. The wedge micromotion changes in edges *aa*, *bb*, and *cc* were regarded as the indices of the plate stability [22, 24]. The micromotions in the initial design plate at *aa*, *bb*, and *cc* were lower than 200 μm except for *cc* in the Puddu plate. Claes et al. reported that a micromotion of 200 μm between segments in a bony structure stimulated callus formation during the early stages of healing [43]. In addition, Pilliar et al. showed a maximum value (> 100 μm) of the allowable movement for the bone union [44]. However, some studies showed that high rigid fixation could lead to osteoporosis because of the stress shielding effect [45, 46]. Moreover, adequate micromotion in fracture interfaces can improve callus formation [47, 48]. Our results showed the best stability with the least micromotion in the TomoFix plate. In a previous study, the least micromotion provides a greater area to cover the circumferential sides of the medial opening in a two-leg plate compared with a locking screw [22, 24]. Previous studies claimed that there are still many arguments regarding the trade-off between rigid fixation and interfacial micromotion [21, 22, 42]. The TomoFix plate provided the greatest stability, but it did not show adequate micromotion which may cause the callus formation problem [8, 42, 45, 46].

The optimal design plate provided the reduced micromotion and improved stability compared with the Puddu plate. Therefore, the optimal design plate suggested the potential to overcome problems in stability and stress shielding with the plate in a trade-off relationship. The optimal design HTO plate provided an environment for successful healing based on the following criteria: plate stress below implant material yield (no permanent deformation of the implant), load sharing without stress shielding on the bone and the plate, and micromotion below 200 μm suggested for callus formation and mineralization.

Relative to a long fixation plate, a short plate was developed with a simple and secure design, leading to the easy achievement of the expected opening amounts on the coronal and sagittal planes, and the facilitation of their combination with other procedures [19]. In addition, a short plate and allogeneic bone graft to treat OA of the knee with a genu varum deformity showed good results with regard to the precision of the correction angle, the time to bone union, and functional improvements based on a previous study [6].

Our results showed that functional improvements in the optimal design short plate were as good as those of the long plate. This was possible through design optimization, and patient-specific design could be possible with various objective functions.

There are several limitations to the present study. First, simulations were performed under only static conditions because the idealistic dynamic motion of the joint was too prohibitive in terms of computing resources and time. In future studies, we may explore a more suitable representation of the joint as well as an analysis of the system under cyclic loading. Second, only the tibia was modeled. Extension of the scope to focus on cartilage or the patella may be required for local mechanical environments to investigate pressing biomechanical questions in HTO. Third, only cortical and cancellous bones were modeled with material properties referred by literature references. In reality, properties of the bone could be varied by the age and history of the disease. Fourth, screw threads are disregarded for numerical simplification, because the purpose of this study was to evaluate plate design. In addition, previous studies also did not represent threads of screw [21, 23, 24]. Fifth, our result cannot be applied to osteoporotic patients and different mechanical axis. However, the purpose of our study was to evaluate the biomechanical effect for the HTO plate design through optimization regardless of bone quality and mechanical axis. Finally, we verified the optimum design plate using a single male subject. However, the purpose of the study was to evaluate improvement in biomechanical effect with the modified design throughout the optimization process. In addition, the advantage of a computational simulation using a single subject is that we could determine the effects of component alignment within the same subject without the effects of variables such as weight, height, bone geometry, and material properties [49]. However, our study has strength over the current computational modeling studies. Screw and bone modeling were bonded in most previous biomechanics study using computational simulation. In this approach, it is beneficial and time-efficient in computational work with no contact condition [21–24, 50]. However, the bonded condition reflected osteointegration in bone, and we aimed to investigate post-operative results in short-term follow-up. Therefore, a more realistic contact condition between screw and bone was applied.

## Conclusions

In conclusion, the results of this preclinical study supposed that the plate design might have a strong influence on the stability after HTO. The TomoFix plate showed better stability compared with the Puddu plate. However, a newly designed plate by an optimization procedure can increase the stability of the implant compared with the initial Puddu plate. This study demonstrated that the optimized short plates had low stress shielding effect and less micromotion because of their improvement in biomechanical performances. This information can aid future developments in HTO plate design and can be expanded to other implant designs.

## Data Availability

Not applicable.

## Abbreviations

3D: Three-dimensional
CT: Computed tomography
DOE: The design of experiments
FE: Finite element
HTO: High tibial osteotomy
NSGA-II: The nondominated sorting genetic algorithm II
OA: Osteoarthritis
